# Interaction of Val66Met Brain-Derived Neurotrophic Factor and 5-HTTLPR Serotonin Transporter Gene Polymorphisms with Lifetime Prevalence of Post-Traumatic Stress Disorder in Primary Care Patients

**DOI:** 10.3390/genes15111355

**Published:** 2024-10-22

**Authors:** Alejandra Guzman-Castillo, Benjamín Vicente, Kristin Schmidt, Esteban Moraga-Escobar, Romina Rojas-Ponce, Paola Lagos, Ximena Macaya, Juan-Luis Castillo-Navarrete

**Affiliations:** 1 Departamento de Ciencias Básicas y Morfología, Facultad de Medicina, Universidad Católica de la Santísima Concepción, Av. Alonso de Ribera 2850, Concepción 4090541, Chile; aleguzman@ucsc.cl; 2 Programa de Neurociencia, Psiquiatría y Salud Mental, NEPSAM (http://nepsam.udec.cl), Universidad de Concepción, Barrio Universitario s/n, Casilla 160-C, Concepción 4070386, Chile; bvicent@udec.cl (B.V.); krisschmidt@udec.cl (K.S.); esteban.moraga.es@gmail.com (E.M.-E.); romrojas@udec.cl (R.R.-P.); ximenacecimacay@udec.cl (X.M.); 3 Departamento de Psiquiatría y Salud Mental, Facultad de Medicina, Universidad de Concepción, Av. Juan Bosco s/n 3er Piso, Box 160-C, Concepción 4070529, Chile; 4 Departamento de Farmacología, Facultad de Ciencias Biológicas, Universidad de Concepción, Barrio Universitario s/n, Box 160-C, Concepción 4070386, Chile; paolalagos@udec.cl; 5 Departamento de Tecnología Médica, Facultad de Medicina, Universidad de Concepción, Barrio Universitario s/n, Box 160-C, Concepción 4070386, Chile

**Keywords:** post-traumatic stress disorder, BDNF Val66Met polymorphism, 5-HTTLPR promoter polymorphism, depressive episode, child maltreatment

## Abstract

**Background/Objectives:** Post-traumatic stress disorder (PTSD) is a complex condition influenced by both genetic and environmental factors. This longitudinal study aimed to explore the connection between two specific genetic polymorphisms, Val66Met and 5-HTTLPR, and the lifetime prevalence of PTSD in patients from primary care settings. We also examined the role of sociodemographic and psychosocial factors to provide a more comprehensive view of PTSD risk. **Methods:** We recruited a cohort of primary care patients and diagnosed PTSD using a standardized diagnostic interview. Genetic analyses focused on Val66Met and 5-HTTLPR polymorphisms. We applied logistic regression to assess the association between these genetic markers and PTSD, considering factors such as gender, family history of depression, and experiences of childhood maltreatment. **Results:** Our findings show that women, individuals with a family history of depression, and those exposed to childhood maltreatment have a higher risk of developing PTSD. While the Val66Met polymorphism was not significantly associated with PTSD, the 5-HTTLPR polymorphism showed a marginal relationship. No significant interaction was found between the two polymorphisms in relation to PTSD. **Conclusions:** This study underscores the multifactorial nature of PTSD, influenced by both genetic and environmental factors. The findings point to the importance of further research on genetic predispositions and highlight the value of early interventions for high-risk populations in primary care settings.

## 1. Introduction

Post-traumatic stress disorder (PTSD) is a chronic disorder that results in significant functional impairment. A significant traumatic event has a substantial impact on the development of PTSD. PTSD symptoms include the re-experiencing of the traumatic event through intrusive thoughts, nightmares, and flashbacks. Furthermore, it presents as negative alterations in cognitive processes and mood, as well as changes in arousal and reactivity [1,2,3,4].

Those with PTSD demonstrate enhanced memory consolidation in relation to fear-related stimuli [1,5]. The neurobiology of PTSD remains a complex field of study. The pathophysiology, progression, and persistence of PTSD are shaped by a multitude of factors. Genetic factors, for instance, have been demonstrated to influence stress responses, with heritability reaching 49% in certain groups [6,7].

Research has identified polymorphisms in genes like Val66Met in the BDNF gene [8,9], 5-HTTLPR [6,10], and the FKBP5 gene that regulates the hypothalamic–pituitary–adrenal (HPA) axis, and these have been linked to the development of PTSD [11]. Brain-derived neurotrophic factor (BDNF) has been demonstrated to modulate a number of key processes within the nervous system, including dendritic branching, spine morphology, synaptic plasticity, and long-term potentiation (LTP). It affects memory, learning, appetite, and sleep [12,13,14]. The BDNF gene has a specific single-nucleotide polymorphism (SNP) at nucleotide 196 (rs6265, G/A), which results in the substitution of valine (Val) for methionine (Met) within the proBDNF 5′ protein at codon 66 (Val66Met) [3,8,14,15]. This alteration affects the packaging of BDNF, resulting in a reduction in its activity dependent on release [8,14].

The Val/Met variant is associated with cognitive deficits, including impaired memory and reduced hippocampal activity [8,16,17]. Furthermore, chronic stress in Met variant carriers may intensify the fear circuit, increasing susceptibility to anxiety and PTSD [3,8,18]. The activity of serotonin transport is subject to influence from a polymorphism linked to the 5-HTT gene, designated 5-HTTLPR (rs25531). This polymorphism is characterized by the presence of 14 or 16 repeat insertions/deletions, which give rise to the formation of two distinct variants: the Long (L) and the Short (S). A single-nucleotide polymorphism (SNP) at position A of the L variant results in the generation of the Lg allele. The Lg and S variants have been demonstrated to reduce 5-HTT gene transcription, which in turn results in a decrease in transporter levels and 5-HT uptake. Conversely, the La variant has been demonstrated to enhance 5-HTTLPR levels, thereby increasing 5-HT uptake [19,20,21,22]. It has been demonstrated that traumatic events can result in an elevation of serotonin release in specific regions of the brain. Individuals who carry the Lg and S alleles may experience difficulties in regulating 5-HTTLPR expression, which could potentially impact extracellular serotonin levels [7,20,23,24]. This can increase the likelihood of developing stress-related disorders, including post-traumatic stress disorder (PTSD). The influence of the Val66Met polymorphism of the BDNF gene and the 5-HTTLPR polymorphism on stress regulation and the risk of developing trauma-related disorders has been identified by research. In light of the aforementioned evidence, we put forth the following hypotheses. Firstly, it is hypothesized that carriers of the Met allele of the Val66Met polymorphism and the S allele of 5-HTTLPR will have an increased risk of developing post-traumatic stress disorder (PTSD) compared to carriers of other genetic variants. Secondly, this risk will be moderated by sociodemographic factors, including gender, family history of depressive episodes, and experiences of childhood maltreatment. Previous studies have demonstrated that these genetic and environmental interactions play a pivotal role in PTSD vulnerability, particularly in populations exposed to traumatic events [19,25,26,27]. Our study aims to explore these associations in a heterogeneous population, thereby providing new insights into the multifactorial risk factors contributing to PTSD development.

## 2. Materials and Methods

### 2.1. Design

This paper presents a longitudinal study of a sample of patients, aged 18 to 75 years, who attended 10 primary care centers in Concepción, Chile. Between 2005 and 2008, the PREDICT-FONDEF study aimed to develop a predictive algorithm for depression based on psychosocial factors in a cohort of primary care patients [28]. The participants were recruited during routine visits to these centers, where eligible individuals were invited to participate in this study. The eligibility criteria included being a patient at one of the selected centers and agreeing to provide informed consent. Efforts were made to ensure a representative sample by including a wide range of sociodemographic backgrounds. Additionally, rigorous follow-up methods were employed to minimize participant attrition over the course of the study.

### 2.2. Participants

**Figure 1:**
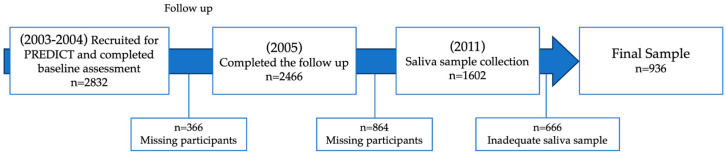
Flow diagram of excluded/ineligible individuals.

The cohort of 936 participants included in this study corresponds to the previously described cohort by Rojas et al. (2015). In 2005, the PREDICT-FONDEF project enrolled 2832 patients for follow-up, of whom 87.1% (n = 2466) completed the 12-month follow-up [25,26]. In 2011, 1602 subjects were contacted and provided saliva samples for genotyping studies. A total of 666 subjects were excluded from the study due to inadequacy of their samples. The resulting final sample consisted of 936 participants (Figure 1).

Participants were excluded from the study if they were unable to understand the local language or if they had a psychotic disorder, dementia, or a physically incapacitating illness. These criteria were selected to ensure that the participants could fully comprehend the nature of the study and provide informed consent. Additionally, individuals with severe mental or physical conditions that could interfere with the accurate assessment of post-traumatic stress disorder (PTSD) and related variables were excluded. Furthermore, only participants who could be located and provided an adequate saliva sample along with informed consent were included in the final analysis, as illustrated in Figure 1.

### 2.3. Instruments

The Spanish-language version 2.1 of the Composite International Diagnostic Interview (CIDI) was used in this study [29]. The CIDI is a structured psychiatric diagnostic tool with good psychometric properties and is widely used [30,31,32]. Additionally, there are no restrictions on its use. The CIDI is conducted by trained lay interviewers without the use of outside sources of information or medical records, minimizing the potential for cultural or regional variations in the perception and documentation of these experiences [29]. The translated version utilized (the official Spanish translation from the World Health Organization) has been validated in Chile [33]. The Depressive Disorders module was utilized to diagnose depressive episodes (DEs). The CIDI provided reliable and standardized assessments of DEs and PTSD, ensuring accuracy and validity. Concerning the sociodemographic information and associated risk factors, we briefly describe how they were obtained.

During the PREDICT-FONDEF study, a comprehensive set of environmental risk factors for PTSD was collected (in individuals without intellectual disabilities). These risk factors were compiled using an inventory from the PREDICT-Europe Project and were also based on known risk factors from earlier literature [28]. This includes valid and reliable self-administered measures. The set of risk factors encompasses demographic factors, family history of psychiatric disorders, DEs, and childhood maltreatment experiences. The latter category includes the death of a family member, physical injuries, and damage or loss of housing.

### 2.4. Ethical Issues

According to the authors, all procedures used in this study and the 2008 revision of the 1975 Helsinki Declaration adhere to the ethical guidelines set forth by the pertinent national and institutional human experimentation committees. All procedures involving patients or human subjects received approval from the Ethics Committee of the Faculty of Medicine at the Universidad de Concepción. An informed consent form was signed by each person who consented to participate, and their data have been fully anonymized and cannot be identified through the manuscript.

### 2.5. DNA Extraction

Saliva samples were obtained, preserved, and transported using a DNA collection kit (Oragene-DNA G-500; DNAgenotek^®^, Stittsville, ON, Canada). DNA was extracted according to the manufacturer’s protocol. The DNA concentration was then quantified using an Infinite^®^ 200 PRO NanoQuant spectrophotometer (Tecan, Männedorf, Switzerland). Finally, DNA integrity was confirmed using agarose gel electrophoresis.

### 2.6. Val66/Met BDNF Genotyping

Val66/Met BDNF was typed using restriction enzyme-based PCR (BsaA I). Specifically, the oligonucleotide partitions, sense F-1F (5′-ATCCCGGTGAAAGAAAGCCCTAAC-3′), and antisense F-1R (5′-CCCCTGCAGCCTTCTTCTTTTGTGTAA-3′) were used to amplify a PCR fragment 673 bp in length. The PCR fragments were then digested with the restriction enzyme BsaA I (New England Biolab, Ipswich, MA, USA). Specifically, this enzyme produces 3 fragments of 275, 321, and 77 bp when guanine is present at nucleotide 1249. In contrast, when cytosine is present at this position, 2 fragments of 321 and 352 bp are produced. Finally, the digested PCR products were separated using a 1.2% agarose gel.

### 2.7. 5-HTTLPR Genotyping

Genotyping of 5-HTTLPR for short and long alleles was performed using PCR [34,35]. These alleles were amplified with the following partitions: sense F1 (5′-TCCTCCGCTTTGGCGCCTCTCTTCG-3′) and antisense R1 (5′-TGGGGGGTTGCAGGGGGGAGATCCTG-3′). These primers produce a 469 bp product for the short allele and a 512 bp product for the long allele. Then, digestion of the PCR fragments was performed using the MspI I restriction enzyme (New England Biolab, Ipswich, MA, USA). As a result, the following cut patterns were obtained: SA—469 bp, SG—402 bp and 67 bp, LA—512 bp, and LG—402 and 110 bp. Finally, these fragments were visualized on a 3% agarose gel. Additionally, all genotyping reactions were performed in duplicate.

### 2.8. Val66Met and 5-HTTLPR Polymorphism Analysis

Comparison groups were established to analyze the impact of these polymorphisms. Additionally, they were used to consider combinations of higher-risk versus lower-risk alleles for developing psychiatric disorders for each gene [36,37]. Thus, those homozygous alleles that would condition lower transcriptional and/or secretory activity are A/A and S/S’ for Val66Met and 5-HTTLPR, respectively. Consequently, the groups at lower risk of developing psychiatric disorders are G/G and L/L for Val66Met and 5-HTTLPR, respectively. Likewise, heterozygotes (G/A and L/S’ for Val66Met and 5-HTTLPR, respectively) were also compared with the higher-risk alleles, as they might also be at risk of developing psychiatric disorders.

### 2.9. Variables

To examine the interaction between various factors and the presence of PTSD, several variables were considered. Demographic confounding variables were obtained from the baseline CIDI assessment, while genetic variables included BDNF and 5-HTTLPR gene variants. A questionnaire created especially for the PREDICT study was used to collect sociodemographic and psychosocial data. This includes a family history of DE and experiences of childhood maltreatment (physical, emotional, and/or sexual). Consequently, the number of maltreatment forms was taken into account, irrespective of their type.

### 2.10. Statistical Analysis

**Figure 2:**
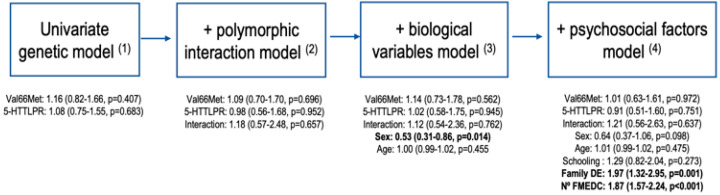
Hierarchical construction of logistic regression models. (1) Val66Met polymorphism (GG/GA-AA); 5-HTTLPR polymorphism (Other/S’/S’); (2) interaction (Val66Met (GA-AA)—5HTTLPR (S’/S’) polymorphic interaction); (3) sex (female/male); age (mean SD); (4) schooling (illiterate, basic, secondary or higher); family DE: family history of depressive episodes (no/yes); Nº FMEDC: number of forms of maltreatment experienced during childhood (mean SD; variable created to group the number of forms of maltreatment experienced in childhood, independent of the type of maltreatment (physical, emotional, or sexual)).

A significance level of *α* = 0.05 was considered for all analyses. Specifically, RStudio version 2.15.2 [38] was used. Using the Kolmogorov–Smirnov and Shapiro–Wilk tests, 913 samples were tested for normality. Between-group differences in categorical variables for those with and without a PTSD diagnosis were calculated using the chi-square test, while differences in continuous variables were calculated using Student’s *t*-test. In the regression analysis, independent associations between genetic predictors and PTSD risk were examined. A univariate logistic regression analysis with a logit link was used to determine odds ratios and 95% confidence intervals. To test the association between all variables (genetic, biological, and psychosocial) and PTSD risk, multivariate logistic regression analyses were performed. These models were built hierarchically based on theoretical reasoning. The first two models included only genetic risk factors, independently and with their interaction. The subsequent model incorporated additional biological variables, followed by a model including psychosocial factors (Figure 2).

## 3. Results

### 3.1. Descriptive Analysis

The sample consisted of 937 people, 78.8% women and 21.2% men (Table 1). Of all the participants, 15.71% developed PTSD during their lifetime, being more frequent in women than in men, with 86.4% and 13.6% (*p* < 0.05), respectively.

Regarding the presence of the Val66Met polymorphism, no statistically significant differences (*p* = 0.707) were found between the groups of participants who developed lifetime PTSD. Heterozygous (G/A) participants numbered 433 (46.53%), and of these, 72 (49.0%) developed lifetime PTSD.

There were also no significant differences for the participants who developed lifetime PTSD according to 5-HTTLPR genetic variants (*p* = 0.415). The L’/S’ and S’/S’ alleles accounted for 93% of the participants with PTSD.

Of all the participants, 24.6% had a family history of depression, which was associated with the presence of lifetime PTSD (*p* < 0.001).

**Figure 3:**
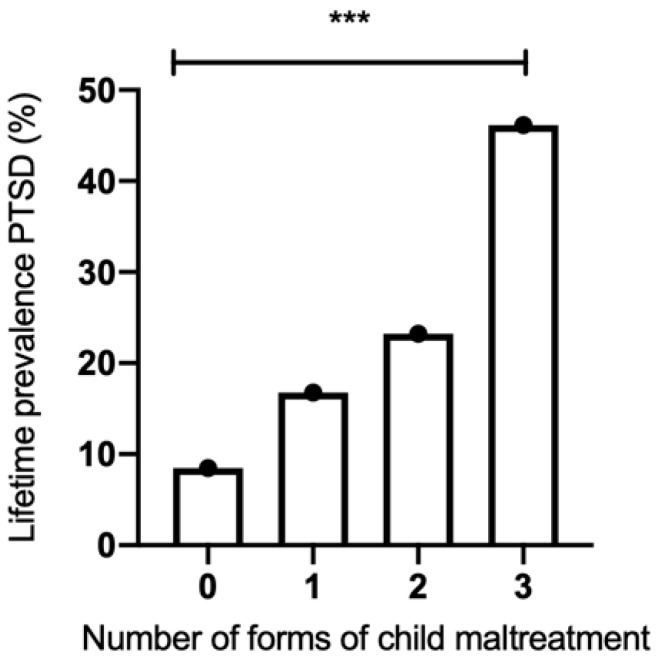
Number of forms of child maltreatment and lifetime prevalence of PTSD; (***) chi-square test (X^2^) = 73.11, df = 3, *p* ≤ 0.0001.

Regarding the number of forms of maltreatment experienced during childhood, 47.1% reported having experienced one or more forms of maltreatment (Figure 3). Of the participants with PTSD, 71.4% experienced some form of maltreatment. In this regard, it is interesting to look at Figure 3, where it is clear (and in agreement with the literature) that the frequency of lifetime PTSD increases as a function of the number of forms of maltreatment in childhood (*p* < 0.001).

### 3.2. Regression Analysis

Regarding the regression analysis on the presence of lifetime PTSD and the associations with the different variables (Table 2), it is pertinent to note that this was performed with increasing complexity. Thus, the univariate genetic analysis for both Val66Met and 5-HTTLPR did not show a direct association with the presence of polymorphisms and the development of lifetime PTSD (*p* = 0.407 and *p* = 0.683, respectively). When considering the interaction between both polymorphisms, no significant association was identified either (*p* = 0.657).

When polymorphisms were considered together with some biological characteristics, a statistically significant association for developing lifetime PTSD was found for sex (*p* = 0.014) but not for age (*p* = 0.455). Thus, being male would be a protective factor for the development of lifetime PTSD, with an OR = 0.53, 95% (CI: 0.31–0.86) *p* = 0.014.

Finally, when psychosocial variables were also considered, significant associations for developing lifetime PTSD were found for a family history of depression (*p* = 0.001) and the number of forms of abuse experienced in childhood (*p* < 0.001). In the case of having a family history of depression, this would be a factor doubling the risk of developing lifetime PTSD with an odds ratio of 1.97 (1.32–2.95) *p* = 0.001. Similarly, the number of forms of abuse experienced in childhood (physical, emotional, and/or sexual) is also a factor that almost doubles the lifetime risk of developing PTSD, with an OR = 1.97, 95% (CI: 1.57–2.24) *p* < 0.001.

## 4. Discussion

Several factors determine susceptibility to the development of PTSD. From this perspective, this study included sociodemographic, psychosocial, and biological factors. Thus, we sought to determine how these factors are associated with the presence of lifetime PTSD. Our results indicate that gender, family history of depression, and several forms of childhood maltreatment are associated with the presence of PTSD. In addition, these variables, together with genetic variables, would confer an increased risk of developing PTSD according to the logistic regression model, OR = 1.87. 95% (CI: 1.57–2.24), *p* < 0.001).

In our study, we describe a significant association between the presence of PTSD in women. Gender differences in PTSD have been attributed to both biological and psychosocial factors, with women exhibiting higher vulnerability due to their exposure to more high-impact traumas and distinct neurobiological responses [39]. This result is not surprising, given the growing evidence that women are more vulnerable to developing PTSD than men [4,40,41]. Research suggests that the lifetime risk of PTSD in women is at least twice that of men [41]. This increased risk has been attributed not only to biological differences but also to variations in socialization processes, childhood experiences, and differential exposure to trauma [42]. This disparity is not due to greater exposure to trauma, but rather to the type of trauma to which women are more exposed, in addition to other psychological, social, and biological factors that may potentially explain women’s greater vulnerability to PTSD [43,44,45].

Child maltreatment, on the other hand, is an act or omission by a caregiver that results in harm or risk of harm to a child. It also includes acts of physical, emotional, sexual abuse and/or neglect [46]. In this sense, in Chile, 73.6% of children suffer physical or emotional violence from their parents or relatives [47], a figure that is quite close to the results of our study. Of all the traumas and adversities to which children are exposed, child maltreatment is among the strongest predictors of PTSD [48,49], a debilitating psychiatric condition that affects up to 37.5% of children exposed to maltreatment [50,51]. The results of this work support this background, as the prevalence of childhood maltreatment experience was found to be 47.1% of the adults included in this study (Table 1).

Consequently, the presence of PTSD was significantly higher in those individuals who presented one or more forms of maltreatment during childhood, a result widely described in the literature [52,53,54] which in our study showed a significant proportional relationship (Figure 3).

Despite the above, given that this study aimed to determine the association between some genetic variants and the lifetime prevalence of PTSD, we limited ourselves to studying the number of childhood experiences of maltreatment and not the types of maltreatment, active and passive, described by the WHO [55]. In this regard, a prospective study differentiated types of maltreatment, including physical, emotional, and sexual abuse and neglect, and their subsequent effects on various mental health disorders. In the case of PTSD, there was an association with all the reported types of abuse; however, the experience of multiple maltreatment and sexual abuse showed a higher level of association [54], which is consistent with our results.

Although child maltreatment is among the strongest predictors of PTSD, less than 40% of children who have ever been maltreated are diagnosed with this disorder, suggesting that exposure to child maltreatment alone is not sufficient to explain this risk [56]. In this sense, factors such as family history of depressive episodes may be involved. Thus, some authors describe that, in particular, the diagnosis of DEs in parents correlates with the occurrence of PTSD in children [57,58]. From this perspective, this is consistent with our findings describing an association of PTSD with a family history of DEs. Furthermore, PTSD and DEs are commonly reported as comorbid disorders and are related to previous exposure to psychological trauma. Additionally, trauma is a complex phenotype, but research suggests that sensitivity to trauma has a genetic and thus heritable basis, which both PTSD and DEs may share [4,59].

Interestingly, genome-wide association studies (GWAS) have identified gene clusters implicated in the diagnosis of PTSD [60,61] and other concomitant disorders such as DEs [60]. However, such studies are still limited, as they are largely oriented towards war veterans, who are mostly male, and replication of these studies in different ethnic populations is still pending.

Consequently, to better understand the genetic factors that may affect susceptibility to PTSD in our study population, we set out to assess whether the presence of BDNF and 5-HTTLPR genetic variants and other psychosocial factors are associated with an increased risk of developing PTSD.

These two genetic factors may interact through their effects on brain regions that are involved in the regulation of emotion and the stress response. BDNF exerts influence over synaptic plasticity and neurogenesis in the hippocampus, which is of paramount importance for contextual fear learning and memory consolidation [62].

In studies involving the Val66Met polymorphism, it has been suggested that substitution for the Met allele results in altered intracellular packaging and regulation of BDNF secretion, resulting in decreased brain levels of this neurotrophin, which would negatively affect cortex-driven fear memory extinction through deficits in LTP [63,64]. The combination of Met allele carrier status, along with other alterations such as decreased prefrontal cortex volume, could confer increased susceptibility to anxiety disorders and negative outcomes following trauma exposure through increased sensitivity to threats [18,65]. Studies of fMRI in healthy individuals report reduced prefrontal cortex and increased amygdala activation in Met allele carriers, but not fear extinction [66]. The relationship between Met allele carrier status, BDNF levels, and depression has been extensively studied, but the interpretation of peripheral BDNF levels remains challenging due to significant methodological variations. Previous research [67] highlights these challenges, suggesting that caution is needed when drawing conclusions about the relationship between peripheral BDNF, depression, and physical health outcomes. Despite this, several meta-analysis studies have found no overall effect of the Met allele on symptoms or correlates of PTSD [36,68,69]. A marginal effect has been described when comparing trauma-exposed subjects with and without PTSD [69]. Despite the above, in this study, we did not find a significant association of the Met allele with the presence of PTSD. This could be explained by several variables not identified in our work, in addition to population differences. It is important to note that our study was conducted in a primary care consultation population, which was mostly female. Consequently, further studies are needed to clarify the possible existence of these associations.

On the other hand, 5-HTTLPR is the most studied genetic polymorphism in psychiatric genetics. Despite this, the evidence is not conclusive on its association with PTSD [36,70]. Nevertheless, the 5-HTTLPR polymorphism has been demonstrated to influence serotonin levels, which in turn affect amygdala reactivity and emotion regulation in the prefrontal cortex [71]. Moreover, it has been postulated that individuals carrying the LG and S alleles may exhibit reduced efficiency in maintaining optimal extracellular serotonin levels, thereby increasing the likelihood of developing stress-related disorders [23,24,72]. Some authors have reported that individuals with at least one of the S alleles are more vulnerable to adverse environments [19,36]. In our study, we did not find a significant association between the LG and S alleles in the presence of PTSD.

It is important to consider the limitations of this study when interpreting the results. Firstly, the potential for interference from different subtypes of depressive episodes (DEs) may have influenced the observed associations, particularly in relation to the Val66Met polymorphism. The heterogeneity of depressive episodes was not fully accounted for, which could affect the interpretation of the genetic findings. Secondly, the present study focused on the number of episodes of childhood maltreatment, without considering the intensity or severity of these experiences. Prior research indicates that the influence of maltreatment on PTSD risk may fluctuate, contingent on the specific type and severity of abuse or neglect. As a result, the lack of differentiation in the intensity of maltreatment in our analysis may limit our understanding of how different forms of childhood adversity contribute to PTSD. Additionally, the majority of the sample was female, which may introduce a gender bias in the results. Given that women are generally more vulnerable to developing PTSD, this gender imbalance could limit the generalizability of the findings to male populations. In addition, although this study included genetic polymorphisms such as Val66Met and 5-HTTLPR, we limited our analysis to the association between these polymorphisms and the presence of PTSD, rather than investigating their association with PTSD severity. Furthermore, additional research is required to confirm these findings in more diverse populations and to examine potential interactions with other genetic and environmental factors. This represents a significant avenue for future research.

In conclusion, this study shows that PTSD is a complex phenomenon for which, according to the final regression model proposed, risk factors include the presence of the Val66Met allele of the BDNF gene, S’S’ alleles of the 5-HTTLPR gene, the female sex, a family history of DEs, and experience of one or more forms of childhood maltreatment; OR = 1.87, 95% (CI: 1.57–2.24, *p* < 0.001).

Finally, these results reaffirm what has been described in the literature regarding the multifactorial approach to mental disorders, including PTSD, highlighting the need to identify these factors promptly for screening of individuals susceptible to developing PTSD to provide preventive or timely management.

## Figures and Tables

**Table 1:** Presence of polymorphisms and sociodemographic and psychosocial characteristics based on lifetime PTSD diagnosis.

			Lifetime PTSD		
Variables		Total	No	Yes	*p* *
		(n = 936)	(n = 789)	(n = 147)	
Polym Val66Met	GG	456 (48.7)	389 (49.3)	67 (45.6)	0.707
	GA	433 (46.3)	361 (45.8)	72 (49.0)	
	AA	47 (5.0)	39 (4.9)	8 (5.4)	
Polym 5HTTLPR	L’/L’	156 (16.7)	137 (17.4)	19 (12.9)	0.415
	L’/S’	450 (48.1)	376 (47.7)	74 (50.3)	
	S’/S’	330 (35.3)	276 (35.0)	54 (36.7)	
Sex	Female	738 (78.8)	611 (77.4)	127 (86.4)	0.020
	Male	198 (21.2)	178 (22.6)	20 (13.6)	
Age	Mean (SD)	46.7 (16.2)	46.6 (16.5)	47.5 (14.9)	0.547
Education	Analphabet	36 (3.8)	32 (4.1)	4 (2.7)	0.080
	Primary	288 (30.8)	233 (29.5)	55 (37.4)	
	Secondary	524 (56.0)	454 (57.5)	70 (47.6)	
	Higher	88 (9.4)	70 (8.9)	18 (12.2)	
Family history of DE	No	706 (75.4)	616 (78.1)	90 (61.2)	<0.001
	Yes	230 (24.6)	173 (21.9)	57 (38.8)	
Maltreatment experienced **	0	495 (52.9)	453 (57.4)	42 (28.6)	<0.001
	1	191 (20.4)	159 (20.2)	32 (21.8)	
	2	185 (19.8)	142 (18.0)	43 (29.3)	
	3	65 (6.9)	35 (4.4)	30 (20.4)	

(*) Chi-square test (X^2^) for categorical variables, *t*-test for numerical variables. (**) Variable created to group the number of forms of maltreatment experienced in childhood, independent of the type of maltreatment (physical, emotional, or sexual). Polym: polymorphisms.

**Table 2:** Logistic regression models for factors associated with the development of PTSD in life.

		Logistic Regression Model *			
Variables		Model 1	Model 2	Model 3	Model 4
Polym Val66Met	GG	-	-	-	-
	GA-AA	1.16 (0.82–1.66, *p* = 0.407)	1.09 (0.70–1.70, *p* = 0.696)	1.14 (0.73–1.78, *p* = 0.562)	1.01 (0.63–1.61, *p* = 0.972)
Polym 5HTTLPR	Other	-	-	-	-
	S’/S’	1.08 (0.75–1.55, *p* = 0.683)	0.98 (0.56–1.68, *p* = 0.952)	1.02 (0.58–1.75, *p* = 0.945)	0.91 (0.51–1.60, *p* = 0.751)
Polym Interaction	Interaction (GA-AA): (S’/S’)		1.18 (0.57–2.48, *p* = 0.657)	1.12 (0.54–2.36, *p* = 0.762)	1.21 (0.56–2.63, *p* = 0.637)
Sex	Female			-	-
	Male			0.53 (0.31–0.86, *p* = 0.014)	0.64 (0.37–1.06, *p* = 0.098)
Age	Mean (SD)			1.00 (0.99–1.02, *p* = 0.455)	1.01 (0.99–1.02, *p* = 0.475)
Education	Secondary				-
	Primary or lower				1.29 (0.82–2.04, *p* = 0.273)
	Higher				1.67 (0.88–3.05, *p* = 0.104)
Family history of DE	No				-
	Yes				1.97 (1.32–2.95, *p* = 0.001)
Matreament experienced **	Mean (SD)				1.87 (1.57–2.24, *p* < 0.001)

(*) Model 1: genetic univariate. Model 2: model 1 plus interaction between polymorphisms. Model 3: model 2 plus sex. Model 4: model 3 plus psychosocial variables. (**) Variable created to group the number of forms of maltreatment experienced in childhood, independent of the type of maltreatment (physical, emotional, or sexual). Polym: polymorphisms.

## Data Availability

The data supporting the findings of this study are not publicly available due to ethical and privacy restrictions related to patient confidentiality. However, anonymized data may be made available from the corresponding author upon reasonable request, subject to institutional ethical approval.
